# Effect of aflatoxin B1 exposure on the progression of depressive-like behavior in rats

**DOI:** 10.3389/fnut.2022.1032810

**Published:** 2022-11-17

**Authors:** Syarminie Subramaniam, Mohd-Redzwan Sabran, Johnson Stanslas, Brian P. Kirby

**Affiliations:** ^1^Department of Nutrition, Faculty of Medicine and Health Sciences, Universiti Putra Malaysia, Serdang, Malaysia; ^2^Pharmacotherapeutics Unit, Department of Medicine, Faculty of Medicine and Health Sciences, Universiti Putra Malaysia, Serdang, Malaysia; ^3^School of Pharmacy and Biomolecular Sciences, RCSI University of Medicine and Health Sciences, Dublin, Ireland

**Keywords:** aflatoxin B1, chronic unpredictable mild stress, sucrose preference, fecal bacterial profile, gut microbiota, depressive-like behavior

## Abstract

While it is well documented that aflatoxin B1 (AFB1); one of the most toxic food contaminants is linked to the development of depression. However, the mechanism on how it affects the gut and brain health leading to depressive-like behavior remains unclear. This study was conducted to determine the effect of AFB1 on the progression of depressive-like behavior. Thirty-two (*n* = 32) male Sprague Dawley rats were randomly allocated into four groups: control, low-dose (5 μg AFB1/kg), high-dose (25 μg AFB1/kg) and positive control group; exposed on chronic unpredictable mild stress (CUMS). After 4 weeks of exposure, sucrose preference test (SPT) and force swim test (FST) were used to measure behavioral despair. Fecal samples were selectively cultured to profile the bacteria. Body weight and relative organs weights were compared among groups. AFB1 and CUMS caused reduction in body weight and food intake as well as increased relative weight of adrenal glands, liver, and brain. Rats in AFB1 and CUMS groups had suppressed sucrose preference and prolonged immobility time in FST, wherein this could indicate anhedonia. Besides, fecal count of *Lactobacillus spp.* was significantly low following AFB1 exposure, with increasing count of *Bifidobacterium spp*, in comparison to the control. Indeed, further biochemical analysis and metagenomic approach are warranted to explore the underlying mechanisms on the role of gut microbiota dysbiosis and dysregulation of gut-brain axis due to AFB1 neurotoxicity on the progression of depressive-like behavior.

## Introduction

Aflatoxins, secondary metabolites produced by certain strains of fungi; *Aspergillus flavus* and *Aspergillus parasiticus*, are the mycotoxins of utmost concern to food safety due to their high toxicity (1). Typically, these fungi contaminate cereal crops such as wheat, walnut, maize, cotton, peanuts and tree nuts (2) or in adverse weather or under poor conditions, aflatoxins are synthesized in a broad range of agricultural commodities such as corn and nuts (3). Aflatoxin contamination of food and feed, is particularly prevalent in tropical and subtropical climates, contributing significantly to the high occurrence of a variety of devastating chronic illnesses and aflatoxicosis outbreaks (4). Approximately more than 14 various chemical forms of aflatoxin are present in nature (5). Of all aflatoxins, aflatoxin B1 (AFB1) is the most potent and carcinogenic and been linked to hepatocellular carcinoma, growth suppression, immune system modulation, and malnutrition (6). The predominant aflatoxins such as B1, B2, G1, and G2 can damage the body *via* respiratory, mucous or cutaneous pathways causing overactivation or inflammatory response (7). Chronic dietary AFB1 exposure is known to have potential in inducing oxidative stress and low-grade inflammation (8). As such, AFB1 is postulated to be a potent naturally occurring food contaminant which can cause chronic stress and neurotoxic processes which consequently leads to depression-like behaviors (9). The well-known mechanism of AFB1 toxicity involves the bioactivation of AFB1 into a highly reactive oxygen species (ROS), AFB1-8,9-epoxide (AFBO) through cytochrome P450 (10). Recently, Huang et al. (11) reported that AFB1 manifests a wide range of cytotoxicity on neuronal cells including ROS accumulation, DNA damage, S-phase arrest, and apoptosis, all of which are key factors for understanding the neurotoxicology of AFB1. Indeed, the neurotoxicity effect of AFBI is linked to the perturbations of microbiome and dysregulation of gut-brain axis. Interestingly, AFB1 also causes gut dysbiosis and disrupt the gut microbiota balance as discussed by Liew and Mohd-Redzwan (12). The link between AFB_1_ and intestinal functionality may involve cytokine upregulation indicating an inflammatory response of the gut (13). It was discovered decades ago that stress, whether early in life or later, may affect the microbial balance of the gut (14). However, there are lack of recent studies showing substantial correlation between the influence of gut microbiome on human behavior. While it is well documented that AFB1 is linked to the development of depression, data on the relationship between AFB1 exposure, neurotoxicity and gut microbiota are still scarce and the mechanism on how these factors can lead to depression remains unclear. Hence, this research aimed to elucidate the effects of AFB1 exposure on the progression of depressive-like behavior in rats.

## Materials and methods

Dried standard AFB_1_ was purchased from Trilogy Analytical Laboratory Inc., Washington, USA. Dimethyl sulfoxide (DMSO, AR Grade) was acquired from Fisher. Sucrose (cat. No. S8501) was used for sucrose preference test; MRS, Bifidobacterium and MacConkey agar were purchased from Sigma-Aldrich Company, USA.

### Animals

Male Sprague Dawley (SD) rats (7–8 weeks old, 250–350 g, *n* = 32) were obtained from Labrat Breeders Farm, Puchong, Selangor, Malaysia. This study was performed at animal research house of Faculty of Medicine and Health Sciences, Universiti Putra Malaysia (UPM). All the rats were caged individually with saw dust bedding. The rats were acclimatized under standard laboratory conditions [12 h light/night cycles (light: 0700–1900 h), 20–25°C, 1-week] prior to the AFB1 exposure and treatments. All rats were given *ad libitum* access to food and water throughout study period. Weight and feed intake were monitored and measured on weekly basis. The use of animal in the present experiment was approved by the Institutional Animal Care and Use Committee UPM (UPM/IACUC/AUP-R025/2020).

### Experimental study

Rats were randomly allocated into four groups; control group: animals were oral gavaged with 10% DMSO only; low-dose and high-dose AFB1 treated groups: oral gavaged with complete dosages of 5 and 25 μg/kg in 10% DMSO, respectively. The positive control group: given chronic unpredictable mild stress (CUMS) to induce persisting stress-related behavioral changes according to protocol described by He et al. (15). The dosages of AFB1 in this study was selected based on previous experiment conducted by Wang et al. (16) and were relevant to AFB1 exposure in humans in developing countries. The treatments were carried out for 5 days/week (17). Body weight and food intake of rats from all groups were recorded on weekly basis using electronic balance (A&D Co., Ltd., Tokyo, Japan). At the end of 4 weeks treatment, fecal samples were collected before behavioral tests such as sucrose preference test (SPT) and force swim test (FST) were conducted. Rats were then anesthetized using ketamine (80 mg/kg) and xylazine (5 mg/kg) mixture and sacrificed *via* cardiac puncture.

### Sucrose preference test

Sucrose preference test was carried out for 4 days, 24 h after collecting fecal and urine sample, according to He et al. (15). From day 1 to day 4, all rats were subjected to adaptive training, with two bottles of water available on day 1 and 2, two bottles of 1% sucrose solution available on day 3. On day 4, one bottle of water and one bottle of 1% sucrose solution were accessible. Following a 12-h fasting, each rat was given 200 mL of water and 200 mL of 1% sucrose solution again; however, the position of the bottles was swapped. The amount of water and sucrose solution consumed were recorded after 1 h and again after 12 h. Sucrose preference percentage (%) was defined as follows:


$$\frac{\mathrm{sucrose}\,\mathrm{solution}\,\mathrm{consumption}\,\left(\text{g}\right)}{\left(\mathrm{sucrose}\,\mathrm{solution}\,\mathrm{consumption}\,\left[\text{g}\right]+\mathrm{water}\,\mathrm{consumption}\,\left[\text{g}\right]\right)}\times 100\%$$

### Force swim test

After 24 h completing SPT, FST was done as previously described by Yankelevitch-Yahav et al. (18) and Shin et al. (19) with slight modifications. Rats were placed individually for 6 min in a vertical glass beaker (height: 50 cm, diameter: 20 cm) containing water (depth: 30 cm) at 23 ± 1°C. The water was changed between rats. A video camera placed on the side of the beaker was used to record the test session. Immobility behavior was defined as minimal movement necessary to keep floating. Rats were considered to immobile when they ceased struggling and remained floating motionless in water. The immobility time was analyzed using a Smart version 2.5 video tracking system (Panlab, Barcelona, Spain). The duration of immobility was recorded during the next 5 min of the total 6 min testing period. Increased immobility time is an index of depressive-like behavior.

### Collection of fecal and organs

Following the 24 h after last day of treatment, all rats were kept individually in metabolic cage for the collection of fecal samples. The fresh fecal sample was cultured immediately. Organs (liver, adrenal glands, and brain) were collected upon euthanization, weighed on an electronic balance and expressed as relative organ weights.

### Determination of fecal bacterial profile

Fresh fecal samples were cultured for *Lactobacillus spp., Bifidobacterium spp*. and *Escherichia coli* on MRS, Bifidobacterium and MacConkey agar, respectively to acquire the colony forming unit (CFU).

### Statistical analysis

Statistical analysis was performed using IBM SPSS Statistics 25.0. One-way and two-way analysis of variance (ANOVA) with Tukey HSD *post-hoc* test were conducted for comparison among multiple groups. Data were presented as mean ± standard deviation (SD), *p* < 0.05 or *p* < 0.01 were considered statistically significant.

## Results and discussion

### Body weight and food intake pattern

**FIGURE 1:**
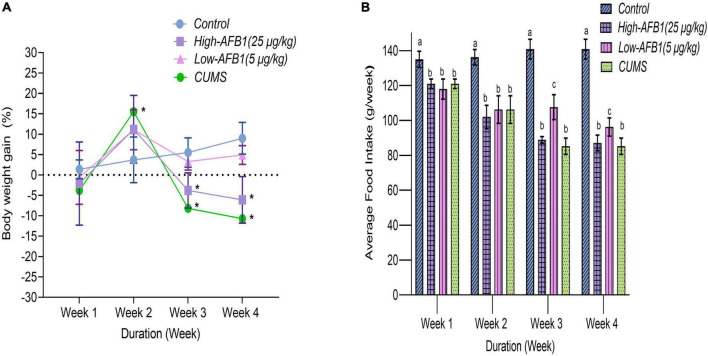
Percentage of body weight gain **(A)** and average food intake in grams per week **(B)** of rats from four different groups during the treatment periods; Week 1 to 4 (*n* = 8). Data are means from eight rats for each group (error bars indicate mean ± SD). In panel **(A)**, asterisk (*) indicates means between different groups within the same week with significant difference (*p* < 0.05). In panel **(B)**, means between different groups within the same week with different lowercase letters (a, b) are significantly different (*p* < 0.05).

Body weight (b.w.) gain and average food intake of rats throughout AFB1 and CUMS exposure period are shown in Figures 1A,B, respectively. In week 1, all groups had an increase of b.w. However, in week 2, 3, and 4, rats exposed to high-dose AFB1 and CUMS had reduction of food intake and experienced statistically significant (*p* < 0.05) weight lost compared to control group. It can be said that the decrease in food intake led to loss of b.w. among rats exposed to AFB1 and CUMS. This showed not only CUMS had an impact on growth, but also AFB1 which exhibited similar pattern. Similarly, Liew et al. (20) reported that growth trajectory patterns of AFB1-exposed rats showed deceleration pattern. It was found that rats treated with AFB1 had lower leptin levels which reduced food intake, subsequently modulated energy balance and body weight (21, 22). According to Saki et al. (23) mycotoxin causes oxidative damage to the cell lining of the GI trat, leading to injury and irritation which reduces nutrient digestion and absorption. This either reduces or slows down process of body weight gain among rats; similar to the trend observed in low-dose AFB1 group in week 3 and 4, although it was not statistically different from the control group. Besides, AFB1 may affect growth performance by causing liver dysfunction and anorexia, as well as *via* inhibition of lipogenesis and protein synthesis as reported by Abbasi et al. (24).

Qiao et al. (25) on the other hand had associated appetite changes; loss or increase and subsequent weight changes with major depressive disorder (MDD). In previous studies involving CUMS model, loss of b.w. in CUMS group was evident by end of week 2 and was persistently lower compared to the control group (15, 26). Lopez et al. (27) stated that CUMS progressively disrupts the metabolism of carbohydrates, lipids, and hormones that control food intake and elucidated the effects of chronic stress on the progression of long-term metabolic disorders. In this study, a dose-dependent decrease of b.w. gain and food intake were found for animals exposed to AFB1 and CUMS. This finding shed light on the effects of chronic stress and AFB_1_ on the progression of long-term metabolic disorders and symptoms of depressive-like behavior.

### Relative organ weights

Organ weight is one of the most sensitive drug toxicity indicators and changes often precede morphological changes (28). As shown in Table 1. AFB1 and CUMS-induced rats had increased relative liver weight. Rats in low-dose AFB1 group had significantly (*p* < 0.05) higher liver-to-body weight ratio (2.90 ± 0.04) than high-dose AFB1 group (2.66 ± 0.09) and CUMS (2.65 ± 0.08). In a study which involved higher dose of AFB1 (75 μg/kg), the authors reported increase in relative liver weight of rats (29). Perhaps, this increase in absolute and relative weights of liver is due to accumulation of lipid in the liver, which produces a characteristic of enlarged and fragile fatty liver (30). It can be postulated that AFB1 may impact the accumulation of lipids and the suppression of lipid transport in the liver, causing an increase in liver weight (31). However, the changes in the liver weight appear to be associated with the AFB1 level in the diet, whereas in the present study, no significant difference in liver weight was discovered in rats fed AFB_1_ at high-dose (25 μg/kg). Indeed, studies by Saminathan et al. (31) and Ma et al. (32) found that the relative liver weights in rats exposed to higher aflatoxin levels (20 and 50–100 μg/kg, respectively) were unaffected.

**TABLE 1:** Relative weights of liver, adrenal glands, and brain of rats in different groups.

Organs	Parameters	Control	High-AFB_1_	Low-AFB_1_	CUMS
Liver	Liver-to-body weight ratio	2.56 ± 0.07^a^	2.66 ± 0.09^a^	2.90 ± 0.04^b^	2.65 ± 0.08^a^
Adrenal gland	AG-to-brain weight ratio	2.40 ± 0.06^a^	2.78 ± 0.17^b^	3.45 ± 0.02^b^	3.28 ± 0.06^b^
Brain	Brain-to-body weight ratio	0.59 ± 0.05^a^	0.68 ± 0.01^bc^	0.61 ± 0.03^ab^	0.71 ± 0.07^c^

Data are expressed as mean ± SD, *n* = 8. Means between groups in the same row that do not share the same lowercase letters (a, b, c) differs significantly (*p* < 0.05) using Tukey HSD *post-hoc* analysis.

In addition, a similar trend was found in the relative weight of AG as the low-dose group showed significantly (*p* < 0.05) higher ratio (3.20 ± 0.43) than high-dose group (2.78 ± 0.17) compared to the control group. Piao et al. (28) indicated that the variation of AG’s weight might be related to the level of hormones secreted by the adrenal zona reticularis. Particularly, higher rate of catecholamine production by adrenals, and possibly the consequential changes in their morphology, were generally associated with stressful conditions (33). Raju and Devegowda (34) noted increased adrenal size with aflatoxin indicated that the toxins induced severe physiological stress in animals. As such, further biochemical tests are recommended to confirm which hormone relatively caused the change in AFB1-induced toxicity in AG. For instance, increased adrenal weight, usually seen in stressed animals, may reflect hyperactivation of the hypothalamic–pituitary–adrenal (HPA) system (35). In this study, CUMS group had the highest significant AG-to-body weight ratio (3.28 ± 0.06). This supports Rygula et al. (36)’s finding whereby rats exposed to chronic stress had significantly increased relative AG weight. Reduced body weight gain and elevated relative AG weights are key indicators of stress exposure in rats (37). It was observed in the present study that AFB1 dosed groups exhibited similar results to CUMS group as both groups showed increase in not only relative AG weight, but also had lost body weight. Hence the changes in the relative AG weight in CUMS and AFB1 dosing groups may be explained through the mechanism of oxidative stress induced by CUMS (38) and also AFB1 through ROS generation (39).

The relative brain weight showed dose-dependent increase as the ratio was higher in high-AFB1 (0.68 ± 0.01) than low-AFB1 (0.62 ± 0.06) group. However, only the high-dose group differed significantly (*p* < 0.05) when compared to control (0.59 ± 0.05) and also, CUMS-induced relative brain weight of rats was significantly the highest (0.71 ± 0.07).

### Fecal bacterial profile

**FIGURE 2:**
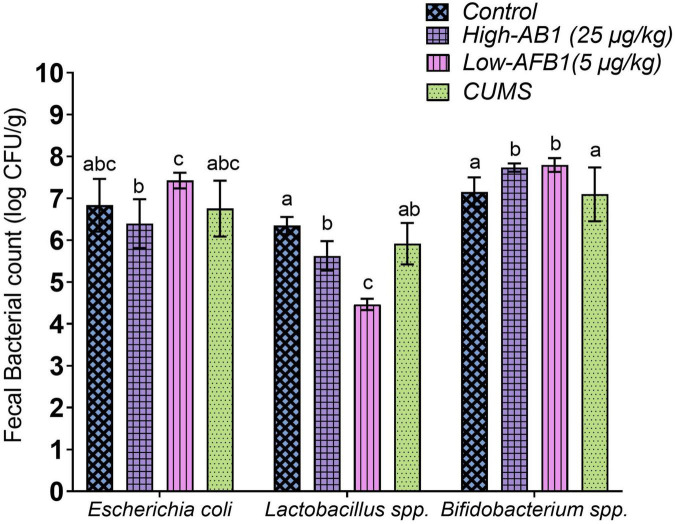
Fecal bacterial count of *Escherichia coli, Lactobacillus* spp., and *Bifidobacterium* spp. Data are expressed as mean ± SD, *n* = 8. Means between groups within columns that do not share the same lowercase letters (a, b, c) are significantly different (*p* < 0.05) using Tukey HSD *post-hoc* analysis.

The count of *Lactobacillus spp*. was significantly (*p* < 0.05) lower following AFB1 exposure in comparison to the control group. Strains of lactic acid bacteria has the ability to bind to toxins such as AFB1 in liquid media and this binding ability could be one of the causes in reduction of its abundance in AFB1 exposed rats. On the other hand, AFB1 exposure reacted inversely with the presence of *Bifidobacterium spp*. as it was significantly higher (*p* < 0.05) than the control group. As for the fecal count of *Escherichia coli*, it was not significantly different (*p* > 0.05) compared to the control group. As for CUMS group, the bacteria were reduced; however, the results were not significant (*p* > 0.05) (Figure 2).

As mentioned earlier, *Lactobacillus spp* reacts with aflatoxins by enzymatic/chemical degradation, metabolic conversion or adsorption through cell wall components (40) and this may have caused the disruption of its presence in the gut. *Lactobacillus spp.* are common probiotics that contribute to the maintenance of intestinal epithelial homeostasis (41), improvement of gastrointestinal barrier function by preventing the proliferation of some harmful bacteria (42) and prevention of chronic inflammatory disease (43). Similar to the current finding, a recent study by Liew et al. (44) also found a decline in *Lactobacillus spp.* upon AFB1 exposure. Cheng et al. (45) classified *Lactobacillus spp.* and *Bifidobacterium spp*. as psychobiotic species because of their mood-enhancing effects. Sarkar et al. (46) further explained that psychobiotic bacteria can alter the microbiota composition under stress and have a beneficial impact on the gut-brain axis in rodent models of psychological stress. Consequently, Aizawa et al. (47) reported a decrease in *Lactobacillus spp.* and *Bifidobacterium spp.* in MDD patients compared with controls. This showed the importance of *Lactobacillus spp*.

Genus of Bifidobacterium is known for its beneficial effects on the host’s health and a lower abundancy is associated with several diseases (48). Nonetheless, AFB1 exposure increased *Bifidobacterium spp*. in this study. Interestingly, previous studies showed an increase in *Bifidobacterium spp*. were mostly associated with depression and negative mood. Most noteworthy, studies by Lai et al. (49) and Rong et al. (50) on MDD patients had shown increased levels of *Bifidobacterium*, which is commonly used as a probiotic. Chung et al. (51) further added that the results of *Bifidobacterium spp.* among MDD patients are ambiguous and the results demonstrated that accumulated internal probiotics may not always translate into good health. Since previous studies have suggested beneficial effect of *Bifidobacterium spp.* and *Lactobacillus spp.* on stress response and depressive disorders (52, 53), both the strains should be considered as adjunctive treatment in the therapy of affective disorder and depressive symptoms (54). In this study, we speculated a change in abundance of these bacteria which may signal a disruption in the intestinal environment.

The difference in the abundance of bacteria from different levels of AFB1 exposure indicated that AFB1 not only damages body organs directly but also disturbs the regular activities of intestinal microflora in animals, as reported by He et al. (55). According to Bharwani et al. (56) stress has a significant impact on the structural and functional features of the gut microflora. Glucocorticoids (e.g., cortisol, corticosterone) secreted during stress, disrupt gut barrier function, lower epithelial integrity, subsequently allow microbes to migrate outside, activating inflammatory immune responses. Bacterial migration outside the lumen could also directly modulate inflammation by increasing pro-inflammatory cell elements such as lipopolysaccharide (LPS), a process associated with human depression (57). Gut dysbiosis is characterized by an increased abundance of proinflammatory species as well as loss of beneficial microbes. Hence, this present study suggests the association between gut microbiome following AFB_1_ and stress exposure in the development of profile similar to depressive-like symptoms.

### Sucrose preference test

The SPT for rodents is based on the animal’s natural preference for sweet substances, with the assumption that this preference is in proportion to the pleasure that the animal experiences. Anhedonia, a core symptom of depression, is the inability to experience pleasure from normally rewarding or enjoyable activities. A reduction in the sucrose preference, in relative to the control group, is indicative of anhedonia (58). In this study, a decrease of sucrose preference (SP) below 65% measured at 4 weeks was taken as a criterion for anhedonia. This criterion was based on the fact that none of the control animals exhibited <65% of SP during the experiment (59).

**FIGURE 3:**
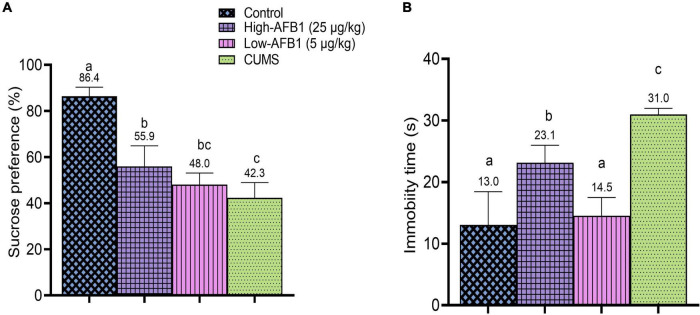
**(A)** Sucrose preference (%) of rats after exposure to AFB_1_ and CUMS treatment. **(B)** Immobility time(s) of rats in FST after exposure to AFB_1_ and CUMS treatment. Error bars represents the data from mean ± SD, *n* = 8. Means between groups that do not share the same lowercase letters (a, b, c) are significantly different (*p* < 0.05) using Tukey HSD *post-hoc* analysis.

In this study, the rats exposed to CUMS had significantly (*p* < 0.05) lower SP (42.3 ± 6.6%) compared to control (86.4 ± 3.9%). The rats in CUMS group were typically affected by the stressors given daily which reflected in their decreased SP. Similarly, CUMS-induced rats in the study by Liao et al. (60) also exhibited reduced SP. Among the AFB1 group, low-dose AFB1 exhibited lower (48.0 ± 5.1%) SP compared to high-dose AFB1 (55.9 ± 9.0%), but the difference was not statistically significant. Hence, it can be postulated that AFB1 exposure produced anhedonia-like behavior among the rats (Figure 3A).

As described earlier, the detoxification of AFB1 produces AFBO, a highly reactive oxygen species (ROS). Excessive production of ROS leads to oxidative stress which has been implicated in the pathogenesis of depressive disorder. ROS can cause enzyme inhibition, lipid peroxidation and mitochondrial alterations, and these are found in depression pathology (61). The current finding shows that rats exposed to AFB1 are experiencing anhedonia-like behavior. Behavioral impairment is often associated with structural and functional changes (62). However, no clear cause-effect relationship between oxidative stress and behavioral alterations could be definitely attained from the present study. As such, more behavioral tests and biochemical studies to assess stress induced by oxidative stress should be performed. These current findings from SPT warrant further investigation on the potential role of oxidative stress on the progression of depressive-like behaviors induced by AFB_1_.

### Force swim test

In the FST, CUMS, and AFB1 significantly (*p* < 0.05) increased the immobility time compared to the control group. As shown in Figure 3B, high-dose AFB1 (23.1s) group showed longer immobility time among rats compared to low-dose (14.5s) and control group (13.0s). Comparatively, increased immobility time seen in high-dose AFB1 corresponds to the CUMS group which exhibited the longest immobility time (31.0s). A study by Wei et al. (63) indicated that CUMS rats were showing behaviors indicative of induced despair/depression proven by the prolonged immobility time in FST.

Furthermore, a recent data by Aytekin et al. (64) reported that AFB1 (25 μg/kg) prolonged immobility time of rats in FST and caused oxidative damage in the brain and triggered inflammatory processes, resulting in anxiety and depression-like behaviors. Oxidative stress plays an essential role in developing behavioral impairment, and depression or anxiety are also related to concomitant oxidative damage and inflammation (65). Afshar et al. (66) further explained that oxidative damage and inflammatory response from oxidative stress also play a crucial role in the toxic effects of AFB1. This could explain the possible effects of AFB1 on the progression of depressive-like behavior in rats which corresponded to the positive control; CUMS group. Indeed, these results confirmed that CUMS rats were showing behaviors indicative of induced despair/depression.

This study shows that AFB1 exerts a significant depressive-like behavior; anhedonia and prolonged immobility time at 5 and 25 μg/kg doses in both the SPT and FST. As such, different doses exert different effect based on SPT and FST results. A possible explanation for the observed dose responses in SPT and FST is the activation of various pathways at different doses.

## Conclusion

This research demonstrated that anhedonia-like behavior, prolonged immobility time indicating behavioral despair, changes in relative weight of brain, liver, AG and fecal bacteria profile were greatly altered following AFB_1_ exposure. Furthermore, the reduction of body weight and food intake among rats correlates with these changes. It is hypothesized that AFB1 induces depressive-like behavior through generation of oxidative stress in brain leading to disruption of HPA axis. At the same time, AFB1 causes gut dysbiosis which can alter HPA axis and affect behavior. However, the mechanism that links both pathways in developing depression remains unclear. Therefore, further histhopathological, biochemical analysis, mechanistic studies as well as microbiota assessment, especially a metagenomic approach, are warranted to delineate the underlying mechanisms. This may possibly shed light on the role of gut microbiota dysbiosis and dysregulation of gut-brain axis due to AFB1 neurotoxicity on the progression of depressive-like behavior.

## Data availability statement

The original contributions presented in this study are included in the article/supplementary material, further inquiries can be directed to the corresponding author.

## Ethics statement

This animal study was reviewed and approved by the Institutional Animal Care and Use Committee UPM (UPM/IACUC/AUP-R025/2020).

## Author contributions

SS: conceptualization, methodology, formal analysis, investigation, data curation, visualization, writing – original draft, and writing – review and editing. M-RS: conceptualization, writing – review and editing, supervision, project administration, and funding acquisition. JS and BK: writing – review and editing and supervision. All authors contributed to the article and approved the submitted version.
